# Challenges When Expanding Transcatheter Aortic Valve Implantation to Younger Patients

**DOI:** 10.3389/fcvm.2018.00045

**Published:** 2018-05-11

**Authors:** Ole De Backer, Lars Søndergaard

**Affiliations:** The Heart Center, Rigshospitalet, Copenhagen, Denmark

**Keywords:** aortic valve stenosis, transcatheter aortic valve implantation, young adults, bicuspid aortic valve, challenges

## Abstract

The rapid expansion of transcatheter aortic valve implantation (TAVI) has been based upon robust clinical evidence derived from randomized controlled trials and large-scale international and national registries. Over the past decade, TAVI has evolved into a safe and effective procedure with predictable and reproducible outcomes. As a consequence, the TAVI technology is increasingly used to treat patients with a lower risk profile and the volume of TAVI now exceeds surgical aortic valve replacement (SAVR) in some countries. It may be anticipated that, in the near future, the majority of patients with severe symptomatic aortic valve stenosis will undergo TAVI as first line therapy, regardless of their age and risk profile. This article identifies some of the specific challenges that lie ahead when considering expansion of TAVI to younger patients.

Transcatheter aortic valve implantation (TAVI) has become an established therapeutic option for patients with symptomatic, severe aortic valve stenosis (AS) who are at increased risk for conventional cardiac surgery (1–4). In recent years, the TAVI technology is also increasingly used to treat patients with a lower risk profile – this practice is supported by results from the NOTION, PARTNER-II and SURTAVI trials indicating that TAVI is a viable option for patients with a low to intermediate surgical risk profile (5–7).

Although TAVI, in many countries, has become the default therapy to treat AS patients aged 75 years or more, there is currently increasing discussion on how far to push the limits when considering treating younger patients. Although the above-mentioned lower-risk TAVI trials included patients with a lower surgical risk score, the mean age of enrolled patients was not different compared to the early TAVI trials conducted in extreme or high risk patients (Figure 1) (1–7). When considering further expansion of TAVI indications to encompass younger patients aged 75 years or less, there are still some challenges ahead.

**Figure 1:**
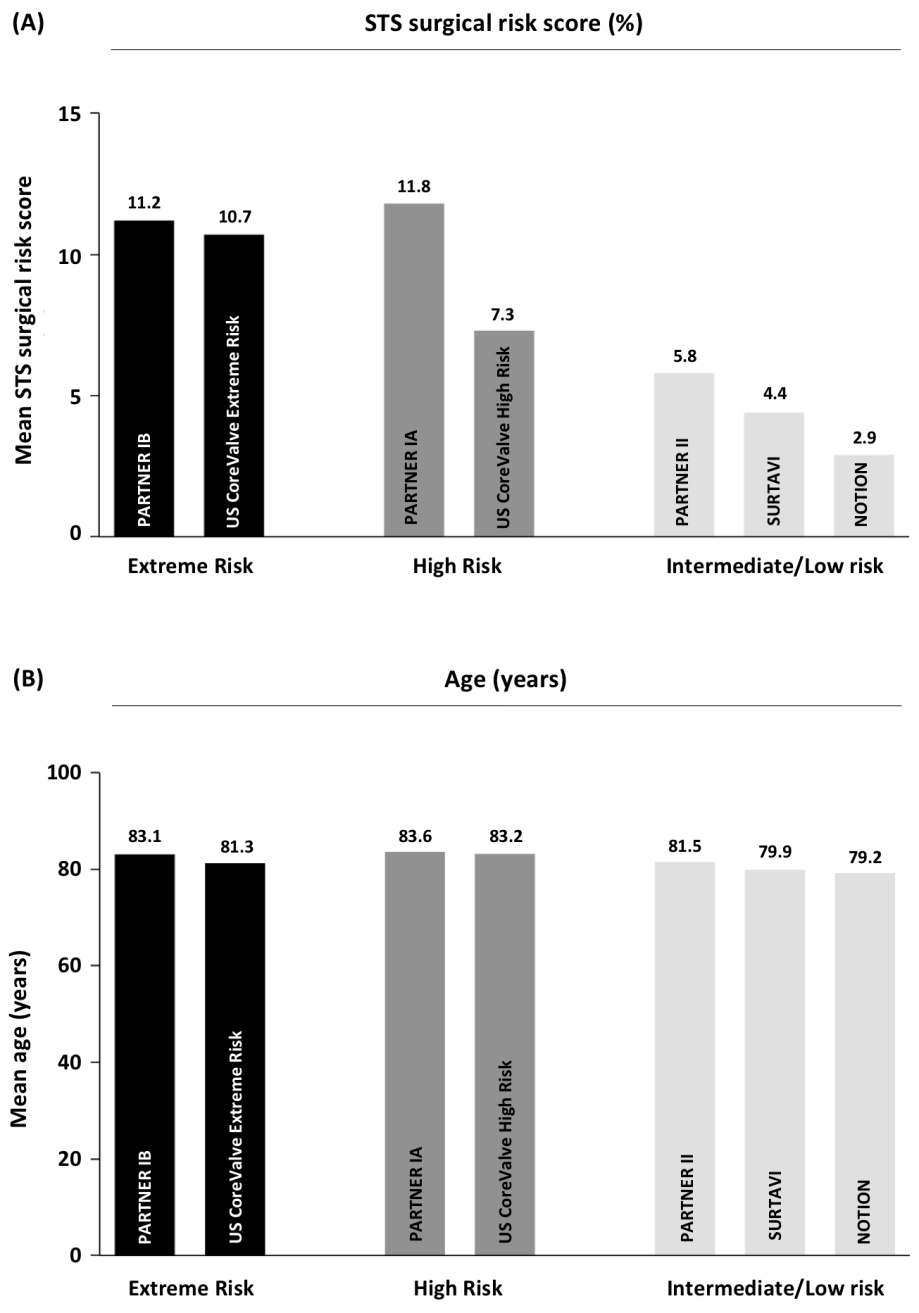
Surgical risk and age profile in the different large randomized controlled TAVI trials, indicating the mean STS surgical risk score (in %) and mean age (in years) for the TAVI group in every respective study. STS, Society of Thoracic Surgeons; TAVI, transcatheter aortic valve implantation.

Based on currently available data, it can be stated that TAVI is non-inferior to surgery in terms of mortality and stroke, and is likely to be superior if a transfemoral approach is possible. Surgical patients more frequently experience major bleedings, acute kidney injury, and new-onset atrial fibrillation, whereas TAVI is associated with a higher rate of major vascular complications, paravalvular regurgitation, and pacemaker implantations (3–7). When considering expansion of TAVI to younger patients < 75 years, it will be a must to obtain predictable and outstanding results, also for these latter procedural outcomes.

Over the past decade, the TAVI technology has matured; however, technological improvements have not come to a halt yet. New TAVI devices with lower-profile delivery systems have increased the proportion of patients who can be treated by transfemoral approach and have significantly reduced vascular complications (8). Newer generation TAVI devices also have an additional sealing skirt, which reduces the risk of paravalvular regurgitation, (9) and are often repositionable, which can result in higher implants thereby reducing the risk of conduction disorders (10). Furthermore, procedural outcomes have improved because of increased operator experience and developments in cardiac and vascular imaging, particularly using multidetector CT.

As TAVI would move into younger AS populations, one pitfall may be that treating bicuspid valves would become an increasing part of practice – with an estimate of 30–50% in those patients aged 75 years or less (11). Importantly, patients with bicuspid AS have typically been excluded from the large randomized controlled trials. Today, only limited data exist on outcomes of TAVI in bicuspid AS. In a recent meta-analysis of 13 observational studies, short-term outcome data indicated that TAVI for bicuspid AS is associated with high device success rates and a good safety profile. Mortality at 30 days was low and comparable to that achieved with the newest generation TAVI devices in tricuspid AS. However, there was a trend towards higher rates of significant paravalvular regurgitation (12%) and permanent pacemaker requirement (18%) in bicuspid AS cohorts undergoing TAVI (12). An important issue when considering TAVI in bicuspid AS is the assessments of these patients’ anatomy and the modification of the TAVI technique, with specific attention to valve deployment and positioning. In order to overcome the limitations of the current generation TAVI devices with regards to paravalvular regurgitation and pacemaker requirement, the design of specific TAVI devices to treat bicuspid anatomy will become crucial.

Finally, extension of TAVI to younger patients with longer life-expectancy also raises the issue of durability. In 2016, some concern was raised about potential poor long-term durability of transcatheter heart valves – however, these results were based on less than 50 first generation valves and only echocardiographic findings were used to define valve degeneration (13) – which is in contrast with the “need for re-intervention” used as definition for surgical valve degeneration. Importantly, since then, robust 5 year follow-up data have come available demonstrating continued valve durability with low rates of hemodynamic valve dysfunction and/or re-intervention, and this for both balloon-expandable and self-expanding transcatheter heart valves (1–3, 14).

Recently, ESC, EAPCI and EACTS have published a consensus on standard definitions of structural valve deterioration (SVD) and bioprosthetic valve failure (BVF) in order to assess long-term durability of transcatheter and surgical aortic bioprosthesis (15). There should be clear distinction between SVD (the principal etiology) and BVF (the clinical correlate). SVD includes permanent (irreversible) intrinsic changes of the valve (i.e., leaflet tear, calcification, pannus deposition, flail, or fibrotic leaflet) leading to degeneration and/or dysfunction, which in turn may result in stenosis or intra-prosthetic regurgitation. The term BVF integrates severe SVD (i.e., the etiology) with its clinical consequences – thereby avoiding over-interpretation of valve-related outcomes in asymptomatic patients with no clinical impact – and is recommended to be used as the main outcome of interest in studies assessing the long-term performance of TAVI and SAVR. Importantly, BVF may occur in the setting of SVD but also as the consequence of pathophysiological processes unrelated to SVD, such as thrombosis, endocarditis or non-structural valve dysfunction. BVF includes any of the following: (1) bioprosthetic valve dysfunction at autopsy, very likely related to the cause of death, or “valve- related death”; (2) aortic valve re-intervention (i.e., valve-in-valve TAVI, paravalvular leak closure or SAVR); and (3) severe hemodynamic SVD.

These definitions have been applied to the NOTION trial (7) – including 80% low risk patients – showing that, after five years, the rate of SVD was lower in transcatheter heart valves as compared to surgical aortic bioprosthesis (3.9% vs. 26.1%, respectively; *p* < 0.001), whereas the rate of BVF was similar in both groups (8.9% vs. 9.5%, respectively; *p* = 0.89) – as presented at EuroPCR 2017.

As a large portion of the younger AS patients has a bicuspid valve, data on transcatheter heart valve durability and long-term outcomes in this specific cohort will also become essential. Given the anatomical characteristics of a stenotic bicuspid aortic valve, a concern may be that the implanted transcatheter heart valve may not fully expand or not become fully circular with asymmetric leaflets as a result. Although this should not necessarily lead to immediate valvular dysfunction, it has recently been reported that asymmetrical leaflet expansion may be associated with an increased risk of subclinical leaflet thrombosis (16). In addition, leaflet asymmetry may also have an impact on long-term valve durability. This is still important missing information when one considers treating a younger bicuspid AS patient with TAVI.

In conclusion, the rapid expansion of TAVI has been based upon robust clinical evidence derived from randomized controlled trials and large-scale international and national registries. Over the past decade, TAVI has evolved into a safe and effective procedure with predictable and reproducible outcomes. As a consequence, the volume of TAVI now exceeds SAVR in some countries (17). It may be anticipated that, in the near future, the majority of patients with severe symptomatic AS will undergo TAVI as first line therapy, regardless of their age and risk profile. This article identifies some of the specific challenges that lie ahead when considering expansion of TAVI to younger patients (Figure 2). With ongoing developments of the TAVI technology, it can be expected that most of these obstacles will be overcome within the next decade. Still, it will be essential to provide the necessary clinical evidence – within the framework of a randomized trial – comparing TAVI with SAVR. Although large TAVI trials have been initiated by Edwards Lifesciences (USA) and Medtronic (USA) in low-risk AS cohorts, this is not a guarantee that young patients will be enrolled. In additon, patients with bicuspid AS are excluded from these trials. Currently, the only large randomized controlled trial comparing TAVI with SAVR in low-risk, younger patients ≤ 75 years of age, not excluding bicuspid valves, is the NOTION-2 trial (ClinTrials.Gov: NCT02825134). This randomized trial should provide the needed clinical evidence to evaluate the use of TAVI in young, low-risk AS patients.

**Figure 2:**
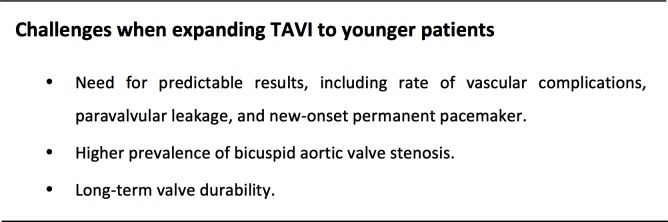
Summary figure: challenges when expanding transcatheter aortic valve implantation to younger patients.

## Author Contributions

ODB and LS both contributed to the concept, writing process and revision of this manuscript.

## Conflicts of Interest Statement

The authors declare that the research was conducted in the absence of any commercial or financial relationships that could be construed as a potential conflict of interest.
